# The Role of *Helicobacter pylori* and NSAIDs in the Pathogenesis of Uncomplicated Duodenal Ulcer

**DOI:** 10.1155/2012/189373

**Published:** 2012-09-25

**Authors:** Ayhan Hilmi Cekin, Muharrem Taskoparan, Adil Duman, Cem Sezer, Yesim Cekin, Basak Oguz Yolcular, Hasan Can, Fatma Seher Pehlivan, Mine Cayirci

**Affiliations:** ^1^Department of Gastroenterology, Antalya Training and Research Hospital, 07050 Antalya, Turkey; ^2^Department of Gastroenterology, Adiyaman State Hospital, Adiyaman, Turkey; ^3^Department of Pathology, Antalya Training and Research Hospital, 07050 Antalya, Turkey; ^4^Department of Microbiology, Antalya Training and Research Hospital, 07050 Antalya, Turkey; ^5^Department of Biostatistics and Medical Informatics, Faculty of Medicine, Akdeniz University, 07050 Antalya, Turkey; ^6^Department of Internal Medicine, Antalya Training and Research Hospital, 07050 Antalya, Turkey; ^7^Department of Pathology, Adiyaman State Hospital, Adiyaman, Turkey

## Abstract

*Background/Aim*. To identify the etiological role of *Helicobacter pylori* (*Hp*) and nonsteroidal anti-inflammatory drugs (NSAIDs) in endoscopically diagnosed duodenal ulcers (DUs). *Methods*. Patients undergoing esophagogastroduodenoscopy in two major hospitals in Antalya and Adiyaman were included in this study and assigned as duodenal ulcer (*n* = 152; median age: 41.0 (16–71) years; 58.6% males) or control group (*n* = 70; median age: 41.0 (18–68) years; 57.1% males). Patient demographics, risk factors, and NSAID/acetylsalicylic acid (ASA) use were recorded. *Results*. *HP* was more commonly located in the corpus (75.0 versus 50.0%; odds ratio [OR] = 3.00; 95% confidence interval [CI]: 1.66–5.44; *P* < 0.001), incisura (75.7 versus 60.0%; OR = 2.07; 95% CI: 1.13–3.79; *P* = 0.017), and antrum (80.3 versus 60.0%; OR = 2.71; 95% CI: 1.45–5.05; *P* = 0.001) among DU patients than controls. *Hp* positivity was 84.9% while *Hp* was negative in 15.1% of patients including those accompanied with NSAID and/or ASA use (9.2%), and those were negative for all three etiological factors (5.9%). *Conclusion*. Our findings indicate the substantial role of *Hp* in the pathogenesis of DU disease as identified in 84.9% of DU patients compatible with the background prevalence of 61.4% among age-matched control subjects. *Hp* was the single causative factor in 44.1% of our patients, while NSAID/ASA exposure was in 9.2%.

## 1. Introduction

The discovery of *Helicobacter pylori* (*Hp*) in 1983, by Warren and Marshall, was one of the most exciting advances in the history of peptic ulcer disease (PUD) which has dramatically changed the management of this clinical entity [1, 2]. Until a few years ago,* Hp* was found to be present in more than 90% of patients with duodenal ulcer (DU) that resulted in the famous dictum “no *Hp*, no DU” [3]. Hence, eradication of *HP* infection even empirically without confirmation of the infection became the mainstay of treatment for PUD resulting in very high ulcer healing rates and a dramatic reduction in recurrence rates [2]. 

However, it has been suggested that the epidemiology of PUD has begun to change dramatically with an increase reported in the proportion of DUs in recent years that are *Hp*-negative [4, 5]. 

Since the prevalence of *Hp*-negative ulcers has been likely to depend upon the background prevalence of *Hp* in the general population, it has been suggested that as the prevalence of *Hp* infection continues to fall over the next decades, the proportion of *Hp*-negative ulcers will progressively increase [2].

In this regard, as demonstrated in 25–75% of the *Hp*-negative DU patients, several observations suggest that non-steroidal anti-inflammatory drugs (NSAIDs) represent the most frequent identifiable cause in DU not associated with *Hp* infection [2, 6–8]. Nonetheless, the precise frequency and causes of *Hp*-negative DUs are still not well known with consideration of lower than previously estimated *H. pylori* infection rates in DU patients by some authors and several hypotheses suggested by others including false negative results because of diagnostic methods, use of NSAIDs and concomitant prescription of proton pump inhibitors (PPIs) [4, 5].

Accordingly, nowadays it is evident that, apart from *Hp* and NSAID usage, there remains a group of patients with ulcers of unknown etiology [4] with arguments put forward to contend against *Hp* as the primary cause of DU disease [9]. Besides, supporting the increasing role of other etiologies in the development of PUD such as NSAIDs and idiopathic ulcers in developed nations, the hospitalization rate for PUD has not shifted despite the decreasing prevalence of *Hp* in Western countries [10]. 

Since most peptic ulcers are caused by *Hp* or NSAIDs, a cause should always be sought with adequate *Hp* testing and careful drug history including over-the-counter medication before the confirmation of *Hp*-negative non-NSAID-associated peptic ulcers [11].

As a matter of fact, given that *Hp* infection remains the most common chronic bacterial infection worldwide, the establishment of a synergistic or additive effect of *Hp* infection and NSAID use in peptic ulcer development has been suggested to be of great clinical importance since eradication of the bacterium would likely reduce the risk of upper gastrointestinal complications in infected NSAID users [12]. 

However, although the presence *Hp* and NSAIDs would be reasonably considered to increase the risk of DU, data from several mainly epidemiologic studies appeared to be controversial and did not always confirm such an assumption [13]. Accordingly the interactions between *Hp* infection and NSAID use in several patient subgroups have not been entirely clarified [14]. 

In view of the significant proportion of *Hp*-negative duodenal ulcers and the possible existence of causal factors other than *Hp*, it is necessary to study the differences between *Hp*-negative and -positive ulcers in order to identify factors other than *Hp*, which are involved in the development of ulcer disease [9]. Besides higher incidence of mortality and recurrent bleeding associated with *Hp*-negative duodenal ulcers were reported to make their documentation important [15]. 

In this regard, while the etiological role of *Hp* as the primary causative factor in DU has been questioned over the past decade, the proportion of *Hp*-negative, NSAID-negative ulcers has not been documented in Turkey. Therefore, based on the crucial role of identification of the etiologic factors associated with ulcer development to establish appropriate management strategies [9], in this study we aimed to identify the etiological role of *Hp* and NSAIDs in endoscopically diagnosed duodenal ulcers and to determine the ratio of the non-*Hp* non-NSAID idiopathic ulcers.

## 2. Materials and Methods

### 2.1. Study Population

Between January 2009 and June 2011, all male and female patients aged 18 years or over who underwent routine endoscopic examination of gastrointestinal symptoms at the Antalya Training and Research Hospital and Adıyaman State Hospital were included in this study and assigned as duodenal ulcer group (*n* = 152) or control group (*n* = 70) based on the presence of endoscopic diagnosis of active duodenal ulcer. An ulcer was defined as a mucosal defect not less than 5 mm in at least one direction. Patients who had prior gastric surgery, cirrhosis, gastric malignancy, end-stage renal disease, inflammatory bowel disease, active gastrointestinal bleeding, *Hp* eradication therapy and treatment with antibiotics, and bismuth-containing compounds within 3 months before the endoscopy were excluded. Also patients who failed to provide an adequate drug history were excluded. Patients consuming PPIs and H2-receptor antagonists were not excluded. Patients (*n* = 70) satisfying the abovementioned criteria but lacking the diagnosis of peptic ulcer disease during endoscopy were assigned as control group if they had been biopsied from gastric antrum, incisura, and corpus for the histopathological diagnosis of *Hp* infection. Written informed consent for upper endoscopy was obtained from all patients before the procedures. Endoscopies were done by experienced gastroenterologists. After endoscopy, participants were interviewed by a doctor to obtain their medical and drug history. Aspirin and NSAID were recorded separately. The study was approved by the clinical research and Ethics Committee of Antalya Training and Research Hospital. 

### 2.2. Diagnosis of *Helicobacter pylori* Infection

During endoscopy, 6 biopsies (two from antrum, two from corpus, one from lover part of lesser curvature for histopathologic examination, and one from antrum again for rapid urease test) were taken. The rapid urease tests (*Hp *fast, GI SUPPLY, Check-Med Systems, Inc. USA) were read at room temperature after four hours. HE and Giemsa staining were performed using serial sections of five specimens from the antrum, corpus, and lover part of the lesser curvature. Results were judged by experienced pathologists who were unaware of other information about patients. Patients were considered to be negative for *Hp* if both histological examination and rapid urease test were negative. Patients were considered positive for *Hp* if any one of the tests was positive.

### 2.3. Statistical Analysis

The *χ* ^2^ test was used for testing associations between qualitative variables; odds ratios (ORs) for significant factors were calculated and presented with 95% confidence interval (CI). Mann-Whitney *U* test was used for comparison of quantitative variables with nonnormal distribution. Results are expressed as *n* (%) or median (minimum-maximum). Data were processed and analyzed with SPSS (Statistical Package for Social Sciences) version 12.0. A value of *P* < 0.05 was considered significant. 

## 3. Results

Control and study groups were homogenous in terms of gender and age with males composing 57.1% of control (median (min–max) age: 41.0 (18–68) years) and 58.6% of DU (median (min–max) age: 41.0 (16–71) years) patients. There was no significant difference between DU and control groups in terms of alcohol consumption (9.9 versus 4.3%), smoking (41.4 versus 34.9%), use of ASA (8.6 versus 14.5%), and the use of NSAIDs (31.4 versus 40.8%) (Table 1).

Comparison of biopsy findings in DU and control groups with respect to location of *Hp *revealed that *Hp* was more commonly located in the corpus (75.0 versus 50.0%; OR = 3.00; 95% CI: 1.66–5.44; *P* < 0.001), the incisura (75.7 versus 60.0%; OR = 2.07; 95% CI: 1.13–3.79; *P* = 0.017), and the antrum (80.3 versus 60.0%; OR = 2.71; 95% CI: 1.45–5.05; *P* = 0.001) regions as well as in any gastric region (84.9 versus 61.4%; OR = 3.52; 95% CI: 1.83–6.76; *P* < 0.001) among DU patients than control patients (Table 2). 

Considering the role of *Hp*, NSAIDs and ASA use in the pathogenesis of the ulcer in DU group, our findings revealed that the ratio of *Hp* positivity was 84.9% including positivity of *Hp* only in 44.1% and positivity of all factors in 40.8% of patients. *Hp* was negative in 15.1% of patients including 9.2% with NSAID and/or ASA use and 5.9% with all etiological factors negative (Table 3).

Patients in control group had antral gastritis (45.7%), reflux esophagitis (24.3%), normal endoscopic findings (15.7%), pangastritis (14.3%), and erosive gastritis (4.3%). 

## 4. Discussion

Our findings related to the etiological role of *Hp* and NSAIDs in endoscopically diagnosed duodenal ulcers revealed the presence of *Hp* in 84.9% of duodenal ulcer patients while in 61.4% of age-matched control subjects. Additionally, among our patients with *Hp*-negative DU, NSAIDs/ASA usage was the underlying cause in 9.2% while non-*Hp* non-NSAIDs/ASA ulcer was evident in 5.9%. 

The prevalence of *Hp* infection has been considered to vary widely both between and within populations based on geography, age, race, ethnicity, and socioeconomic status. Since inadequate sanitation practices, low social class, crowded or high-density living conditions, and inadequate nutritional status were associated with a higher prevalence of infection [16], the rate of acquisition was generally higher in developing countries than in industrialized countries [10]. 

Accordingly, the prevalence of *Hp* infection was reported to be 84% in studies published from 1999 to 2003, while to be 77.2% when only a more recent study period from 2004 to 2008 was considered. Specifically, prevalence of *Hp* infection in DU disease in those studies performed in Europe (83.9%) was higher than in those conducted in USA (72.4%), while intermediate prevalence was described in other American countries such as Brazil, Colombia, and Peru (81.9%), and the highest rates of infection were reported in Japan (94.3%) [2].

In this regard, identification of *Hp* in any gastric location in 84.9% of our study population seems quite comparable to data from European studies and a recent meta-analysis including a total of 16 080 patients indicating the prevalence of 81.2% [2].

Although *Hp* infection and NSAIDs have for long been considered to be the major etiological factors in the causation of PUD, recent years have witnessed a paradigm shift in the epidemiology of the disease with an increase in proportion of *Hp*-negative PUD in developed countries [15].

Recent North American data suggests that up to 50% of ulcers are *Hp* negative [17], while the background prevalence of *Hp* infection has been documented to vary markedly between different countries, being almost 100% in some regions of the developing world and less than 10% in highly affluent communities [18]. Hence identification of *Hp* in any gastric location among 61.4% of age-matched control subjects (mean 42 years of age) in our study indicates high background prevalence of nonulcer *Hp* in our population unlike to decreasing prevalence reported in recent decades in the Western population linked to improvements in living conditions [19].

Likewise, the prevalence of ulcers associated with *Hp* alone, with the use of NSAIDs alone and not associated with *Hp* or the use of NSAIDs, were reported to be 66%, 8.5% and 17%, respectively in a past study conducted with 599 patients with active DU and known *Hp* status [20]. Additionally, evaluation of prevalence and causes of DU in 774 patients [20] revealed *Hp* negativity in 4.6% of cases which was associated with taking NSAIDs in 55.6%, receiving antibiotics in 25.0% with consideration of ulcer as truly idiopathic only in 0.8%. Also, evaluation of 464 patients with endoscopically confirmed DU revealed that *Hp* negativity was reported in 3% associated with taking NSAIDs in 21.4% whereas no apparent explanation for DU (idiopathic DU) was evident in 1.3% [21].

In this regard, given that the proportion of *Hp*-negative ulcers would be only 5% in a country with a 65% background prevalence of the infection [18], our findings indicating background prevalence of *Hp* infection to be 61.4% seem to be consistent with *Hp* positivity of 84.9% as well as non- *Hp* non-NSAID/ASA ulcers in 5.9% of our DU patients. Likewise, the background prevalence of *Hp* infection in Scotland was reported to be 65%, consistent with 95% of the ulcers occurring in *Hp*-infected subjects [22]. 

In fact, markedly higher prevalence of *Hp* among our DU patients compared with their age-matched controls as detected from gastric corpus (75 versus 50%), incisura (75.7 versus 60%), and antrum (80.3 versus 60%), as well as biopsies from any gastric location (84.9% versus 61.4%) supports previous studies indicating that more than 90% of patients with duodenal ulcer are infected with *Hp* [1] and provide a meaningful data on the effect of *Hp* on the risk of DU.

Additionally, given the fact that gastric cancer is more frequent in the eastern populations like in the Turkish population and still has an important impact on health all over the world [23], identification of the antrum as the location of *Hp* in 80.0% of our patients is worth noting in relation to its association with gastric tumor development.

Alike to our findings, in a systematic review [12] concerning effects of *Hp* and NSAIDs on PUD, in 21 studies involving 10,146 patients, uncomplicated peptic ulcer was reported to be more common in *Hp*-positive than *Hp*-negative patients (pooled odds ratio [OR], 2.17) or in *Hp*-positive than *Hp*-negative NSAID users (OR, 1.81).

Besides in 6 age-matched controlled studies, ulcer was reported to be more common in *Hp*-positive than *Hp*-negative patients (OR, 4.03), irrespective of NSAID use, and in NSAID users than nonusers (OR, 3.10), irrespective of *Hp* status while the risk of ulcer was reported to be 17.54-fold higher in *Hp*-positive NSAID users than *Hp*-negative nonusers [12].

Indeed the risk of ulcer was reported to increase 10-fold by the simultaneous presence of *Hp* and NSAIDs positivity compared with the absence of both factors [12]. Hence, our findings related to identification of *Hp* per se as the sole etiologic factor in 44.1% of duodenal ulcer patients while simultaneous presence of *Hp* and NSAID and/or ASA use only in 40.8% of ulcer patients may be associated with the exclusion of patients with active gastrointestinal bleeding since the presence of both factors was detected significantly more frequently in patients with ulcer bleeding than in nonbleeding control subjects [12]. 

Nevertheless, while there is considerable variation in their reported proportions, some ulcers are apparently unrelated to risk factors including *Hp* infection and the use of NSAIDs or ASA [18]. While recent reports from North America suggest that up to 50% of ulcers are *Hp* negative [17, 24], the ratio of *Hp*-negative NSAID-absent ulcers in our study population (5.9%) supports that true idiopathic DU disease only exceptionally exists with the recently reported increase in the prevalence without exceeding 10% [18, 25].

 Indeed while NSAIDs has been the major underlying factor in *Hp*-negative ulcers, several other hypotheses have also been suggested to explain the pathogenesis of *Hp*-negative DU including false negative results due to diagnostic methods, bleeding peptic ulcers, gastric outlet obstruction, perforated peptic ulcers, tobacco use, isolated *Hp* duodenal colonization, age, history of PUD, race, gastric hypersecretion, genetics, diseases of the duodenal mucosa, *Helicobacter* “heilmanii” infection and concomitant diseases [2].

In this respect, identification of *Hp*-negative ulcers in 15.1% of our patients must be interpreted in the light of characteristics of our study population involving relatively younger patients but not patients with concomitant diseases and complicated clinical presentation.

Considering the role of NSAID use in *Hp* negative DU cases, while the duodenal ulcer diagnosis was definitely associated with *Hp* infection in 84.9% of our cases whether as a single pathogenic factor (44.1%) or in combination with ASA and/or NSAIDs (40.8%), *Hp* negative but NSAID and/or ASA-positive ulcers were evident only in 9.2% of our patients. In one hand, this finding is in line with past studies indicating 8.5% [26] and 13% [9] of patients with *Hp*-negative duodenal ulcer to have taken NSAIDs, while on the other hand, much higher prevalence such as 50% [27] and 70% [7] was also reported in the literature for the prevalence of *Hp*-negative DU patients.

Indeed, the background prevalence of *Hp* infection in the community being studied, and the robustness of the exclusion of *Hp* infection and of the use of NSAIDs and aspirin become the two factors suggested to explain this marked variation in the reported proportion of ulcers being unrelated to *Hp* infection and NSAIDs [18].

Nonetheless, based on the published reports stating the use of NSAIDs to be the major cause of and an independent factor associated with *Hp*-negative ulcer disease [1, 9], a careful history and examination of recent prescription records seems necessary to be certain that the patient is not taking unrecognized NSAIDs as well as over-the-counter medications including herbal medications, some of which contain salicylates [18]. 

Moreover, several medications frequently prescribed for patients with upper gastrointestinal (GI) symptoms or disease were reported to result in false-negative *Hp* tests [18], and *Hp* infection was reported to be particularly likely to be missed in a patient with acute upper GI bleeding secondary to ulcer disease [28]. Hence, while patients with *Hp* eradication therapy and treatment with antibiotics, bismuth-containing compounds within 3 months before the endoscopy, as well as acute GI bleeding were excluded in our study, production of false-negative urease tests cannot be disregarded due to inclusion of patients consuming PPIs and H2-receptor antagonists which were also associated with false-negative urease tests by reducing bacterium's urease activity via elevation in the intragastric pH [18, 29]. 

Missing *Hp* diagnosis is considered an extremely important in patients with ulcer disease, as this will deny them the chance of long-term cure of the condition by eradicating the infection and leave them at increased risk of developing ulcer complications over subsequent years [18]. Accordingly, it is important to note that, to ensure that *Hp* infection is not missed, gastric biopsies were taken from both the antrum, corpus, and incisura regions of the stomach in our study.

As a matter of fact, it should be noted that the relative proportion of non-*Hp*, non-NSAID ulcers is expected to increase following progressively decreasing prevalence of *H. pylori* infection whereas underreporting of NSAID use and false positive endoscopic findings should also be taken into account [12]. Therefore, appropriate insurance of the absence of these common risk factors has been indicated as the most important clinical point for *Hp*-negative NSAID-absent ulcers [18]. In this regard it is obvious that all the above investigations regarding other possible causes of duodenal ulcer must be completed before concluding that *Hp*/ASA/NSAIDs-negative duodenal ulcers were evident.

Nevertheless, since the prevalence of idiopathic peptic ulcer which cannot be attributed to either *Hp* or NSAID was reported to remain stable despite a decrease in *Hp*-associated PUD and an increase in NSAID-associated PUD, it has been suggested that duodenal ulcer might persist as a health problem unless other etiologic preventable factors are identified [9].

In conclusion, our findings indicate the substantial role of *Hp* in pathogenesis of duodenal ulcer disease as identified in 84.9% of duodenal ulcer patients compatible with the background prevalence of 61.4% among age-matched control subjects. Based on the diagnosis of *Hp* as a single causative factor in 44.1% of our patients while that of NSAIDs/ASA in 9.2% of cases, *Hp* infection and NSAID/ASA usage seem to represent independent synergistic risk factors for uncomplicated peptic ulcers with possible beneficial effect of *Hp* eradication in NSAID users. Albeit identification of non-*Hp* non-NSAIDs ulcers in only 5.9% of our population supports that true idiopathic DU disease only exceptionally exists, being restricted to data from uncomplicated ulcer cases excluding concomitant diseases and complicated clinical presentation, one has to be cautious when drawing conclusions comparable with a cross-section of the general population based on our findings.

## Figures and Tables

**Table 1:** Demographic features and risk in the study groups.

	Control (*n* = 70)	Duodenal ulcer (*n* = 152)	*P* value
	Median (min–max)		
Age (years)	41.0 (18–68)	41.0 (16–71)	0.342^a^
	*n* (%)		
Male gender	40 (57.1)	89 (58.6)	0.843^b^
Alcohol consumption	3 (4.3)	15 (9.9)	0.157^b^
Smoking	29 (41.4)	53 (34.9)	0.347^b^
ASA use	6 (8.6)	22 (14.5)	0.218^b^
NSAID use	22 (31.4)	62 (40.8)	0.181^b^

ASA: acetylsalicylic acid; NSAID: nonsteroidal anti-inflammatory drugs; ^a^Mann-Whitney *U* test; ^b^Chi-Square *z* test.

**Table 2:** Biopsy findings related to gastric location of *H.  pylori*.

Location of Hp	Control (*n* = 70)	Duodenal ulcer (*n* = 152)	OR (95% CI)	*P* value*
	*n* (%)			
Anywhere	43 (61.4)	129 (84.9)	3.52 (1.83–6.76)	<0.001
Corpus	35 (50.0)	114 (75.0)	3.00 (1.66–5.44)	<0.001
Incisura	42 (60.0)	115 (75.7)	2.07 (1.13–3.79)	0.017
Antrum	42 (60.0)	122 (80.3)	2.71 (1.45–5.05)	0.001

*Hp*: *Helicobacter pylori*; CI: Confidence interval; OR: Odds ratio.

**Table 3:** Pathogenesis of ulcer in the duodenal ulcer group (*n* = 152).

Hp-positive patients (*n* = 129)	*n* (%)
Only HP-positive	67 (44.1)
HP positive *+ *NSAID and/or ASA exposure	62 (40.8)

Hp negative patients (*n* = 23)	

NSAID and/or ASA exposure positive	14 (9.2)
NSAID and ASA exposure negative	9 (5.9)

ASA: acetylsalicylic acid; *Hp*: *Helicobacter pylori*; NSAID: nonsteroid anti-inflammatory drug.
